# Concordance in Radiological Parameters of Different Knee Views After Total Knee Arthroplasty

**DOI:** 10.7759/cureus.38129

**Published:** 2023-04-25

**Authors:** Maximiliano Barahona, Mauricio A Guzman, Felipe Bustos, Gaspar Rojas, Marcela Ramirez, Daniel Palma, Martin Guzman, Macarena A Barahona, Alex Zelaya

**Affiliations:** 1 Orthopaedics, Hospital Clinico Universidad de Chile, Santiago, CHL; 2 Orthopaedics, Clinica Las Condes, Santiago, CHL; 3 Radiology, Hospital Clinico Universidad de Chile, Santiago, CHL

**Keywords:** patient, satisfaction, knee replacement, radiograph, total knee arthroplasty

## Abstract

Background

Total knee arthroplasty (TKA) is a cost-effective treatment for the end-stage of knee osteoarthritis. Despite the improvements in this surgery, a significant percentage of patients still report dissatisfaction after knee arthroplasty. Radiological results have been used to predict clinical outcomes and satisfaction after knee replacement. This study aims to evaluate the concordance of a set of radiographic views to assess alignment on total knee arthroplasty.

Methods

A concordance study was designed with 105 patients (130 TKA) that underwent conventional total knee arthroplasty cruciate-retaining design recruited for the study and scheduled for their annual radiograph control. Measurements were performed on the following radiograph after total knee replacement: full-length standing anteroposterior and lateral radiograph, anteroposterior standing, lateral and axial knee view, and the knee "seated view". A musculoskeletal radiologist and a knee surgeon were recruited to perform the radiological measurement and then estimate the interobserver agreement.

Results

There was an excellent correlation between Limb Length (LL), Hip-knee-ankle angle (HKA), Sagittal mechanical tibial component alignment (smTA), extension lateral and medial joint space (eLJS and eMJS), 90º flexion lateral and medial joint space (fLJS and fMJS) and Sagittal anatomic lateral view tibial component alignment (saLTA); the good correlation between Mechanical lateral femoral component alignment (mLFA), Sagittal anatomic tibial component alignment (saTA), Sagittal anatomic lateral view femoral component alignment 2 (saLFA2), Patella Height (PH); and moderate to poor correlation for the rest of measurements.

Conclusion

Excellent and good concordance can be achieved for radiographic measurements in different knee views to assess results after TKA. These findings must encourage future studies to address functional and survival outcomes using all knee views and not just one plane.

## Introduction

Knee arthroplasty is a cost-effective treatment for the end-stage of knee osteoarthritis. Despite improvements in pain management, new prosthesis design, different philosophies in frontal alignment, the introduction of robotic-assisted surgery, and emphasis on rehabilitation, dissatisfaction after knee arthroplasty remains around 15 to 20% [1].

Some studies have focused on radiological results to predict clinical outcomes and satisfaction after knee replacement [2-4], especially those aiming to compare robotic-assisted surgery and conventional surgery [5, 6]. Nevertheless, these studies focus mainly on anteroposterior views, sometimes lateral views, but less frequently on the axial view of the patella or the rotation of the femoral component. [7, 8]. For example, Keshmiri et al. [9] found that one-degree changes in femoral flexion or tibial slope cause changes in patellar tracking, while Kim et al. [7] describe that in patients with patellar replacement, the component rotation has an inverse correlation with patellar tilt. Therefore, it is necessary to evaluate the outcome of knee arthroplasty by taking measurements from different acquisitions and seeking associations of these measures as a whole with patients’ reported functional outcomes. The first step towards this is determining the degree of agreement among observers for each measurement.

This study aimed to evaluate the concordance of a set of radiological parameters on different radiograph views to assess alignment on total knee arthroplasty.

## Materials and methods

A concordance study was designed with the approval of the ethics committee board of our institution (Hospital Clinico Universidad de Chile, 63/2023). Inclusion criteria were patients that underwent conventional total knee arthroplasty with a Vanguard (Zimmer-Biomet) cruciate-retaining anterior stabilized (CRAS) design without patellar resurfacing. Exclusion criteria were posterior stabilized or revision prosthesis design, less than one year after surgery, and refusal to participate in the study.

Routine radiographs set for knee replacement follow-up in our institution are full-length standing anteroposterior radiographs of the lower extremity, anteroposterior standing knee view, and lateral knee view. A total of 105 patients/ 130 TKA were recruited for the study and were scheduled for their annual radiograph control, signing informed consent to perform more radiograph views than usual. These additional views were: a full-length standing lateral radiograph of the lower extremity, a patellar axial view with the knee flexed at 30º and a posteroanterior view with the patient seated and the knee in 90º of flexion, "the seated view" [10].

The following 32 radiological parameters were assessed for concordance:

Full-length standing anteroposterior radiograph

Right and Left Limb length (LL)

**Figure 1:**
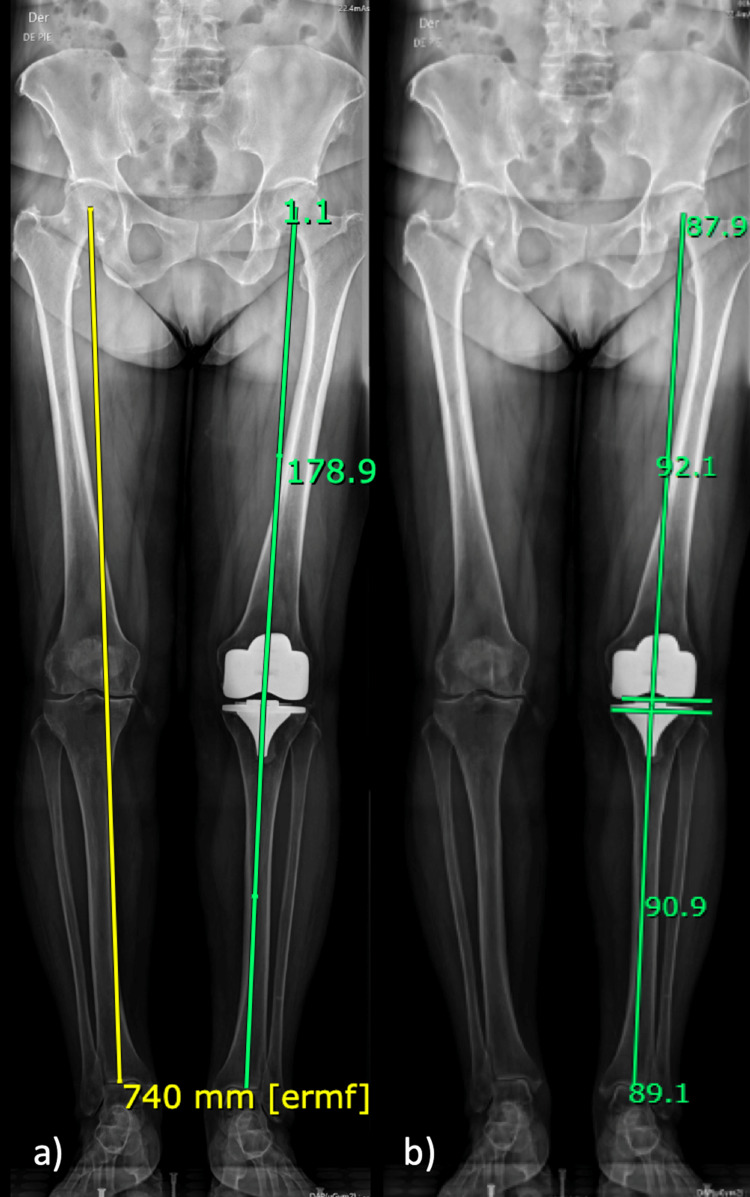
Full-length standing anteroposterior radiograph (a) Limb length (yellow line), the hip-knee-ankle angle (green line); (b) Mechanical lateral femoral component alignment, and mechanical medial tibial component alignment.

Distance from the center of the hip and the center of the ankle, both sides were assessed (Figure 1a).

The Hip-Knee-Ankle Angle (HKA)

The angle was measured between a line from the center of the hip to the center of the tibial component and a line between the center of the tibial component and the center of the angle. A positive angle was used for varus alignment, and a negative in the case of valgus alignment (Figure 1a).

Mechanical Lateral Femoral Component Alignment (mLFA)

The angle was measured between a line from the center of the hip to the center of the femoral component and a line that goes through the most distal part of the lateral and medial femoral components. The lateral angle was registered (Figure 1b).

Mechanical Medial Tibial Component Alignment (mMTA)

The angle was measured between a line from the center of the tibial component to the center of the ankle and a line parallel to the tibial component. The medial angle was registered (Figure 1c).

Full-length standing lateral radiograph

Sagittal Mechanical Femoral Component Alignment 1 (smFA1)

**Figure 2:**
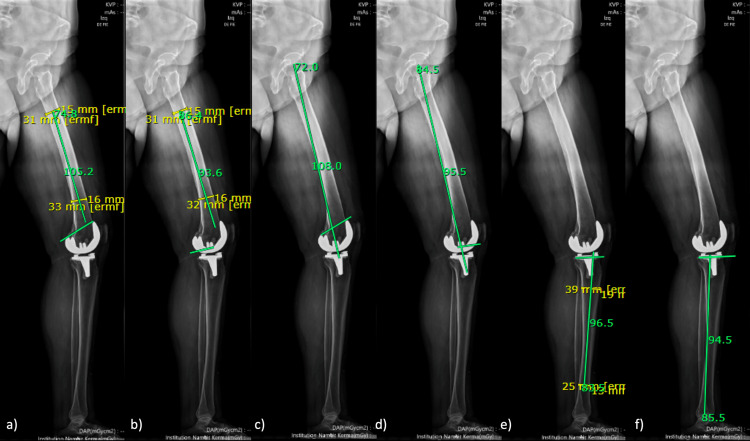
Full-length standing lateral radiograph (a)Sagittal anatomic femoral component alignment 1; (b) Sagittal anatomic femoral component alignment 2; (c) Sagittal mechanical femoral component alignment 1; (d) Sagittal mechanical femoral component alignment 2; (e) Sagittal anatomic tibial component alignment; (f) Sagittal mechanical tibial component alignment

The angle was measured between a line from the center of the hip and a line that goes through the most proximal parts of the anterior and posterior femoral components (Figure 2a).

Sagittal Mechanical Femoral Component Alignment 2 (smFA2)

The angle was measured between a line from the center of the hip and a line that goes through the most distal axes of the femoral component (Figure 2b).

Sagittal Anatomic Femoral Component Alignment 1 (saFA1)

First, in the distal middle shaft of the femur, two lines were drawn from the anterior to the posterior cortex, and the middle of them was highlighted. The angle was measured with a line that goes through the middle of the two lines of the shaft and a line that goes through the most proximal parts of the anterior and posterior femoral components (Figure 2c).

Sagittal Anatomic Femoral Component Alignment 2 (saFA2)

First, in the distal middle shaft of the femur, two lines were drawn from the anterior to the posterior cortex, and the middle of them was highlighted. The angle was measured with a line that goes through the middle of the two lines of the shaft and a line that goes through the most distal axes of the femoral component (Figure 2d).

Sagittal Mechanical Tibial Component Alignment (smTA)

The angle was measured between a line from the center of the ankle to the center of the tibial component and a line parallel to the long axis of the tibial component. The posterior aspect of the angle was recorded (Figure 2e).

Sagittal Anatomic Tibial Component Alignment (saTA)

The first two lines were drawn from the anterior to the posterior cortex of the tibial shaft, one of them on the proximal half of the shaft and the other in the distal half. The middle portion of both lines where highlighted. The angle was measured between a line that connected both half distances of the lines drawn on the tibial shaft and a line parallel to the long axis of the tibial component. The posterior aspect of the angle was recorded (Figure 2f).

Anteroposterior standing knee view

Extension Lateral and Medial Joint Space (eLJS and eMJS)

**Figure 3:**
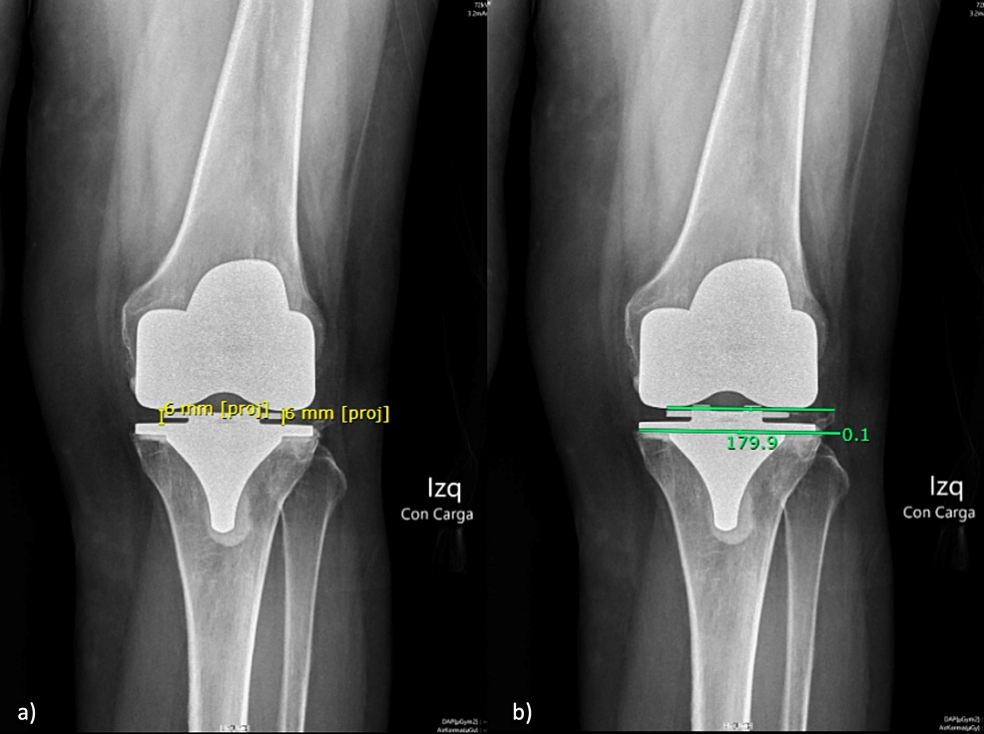
Anteroposterior standing knee view (a) Extension lateral and medial joint space; (b) Extension joint angle

Two distances were registered in millimeters between the most distal part of the femoral component and the tibial component on the lateral and medial sides. Also, the side-to-side difference was calculated (AGext) (Figure 3a).

Extension Joint Angle (eJA)

The angle was measured between a line that joins the most distal part of the medial and lateral femoral component and a line parallel to the tibial component. A positive angle was registered when the lines were medially united, and a negative value was when the lines united on the lateral side (Figure 3b).

Seated view 

Ninety Degree (90º) Flexion Lateral and Medial Joint Space (fLJS and fMJS)

**Figure 4:**
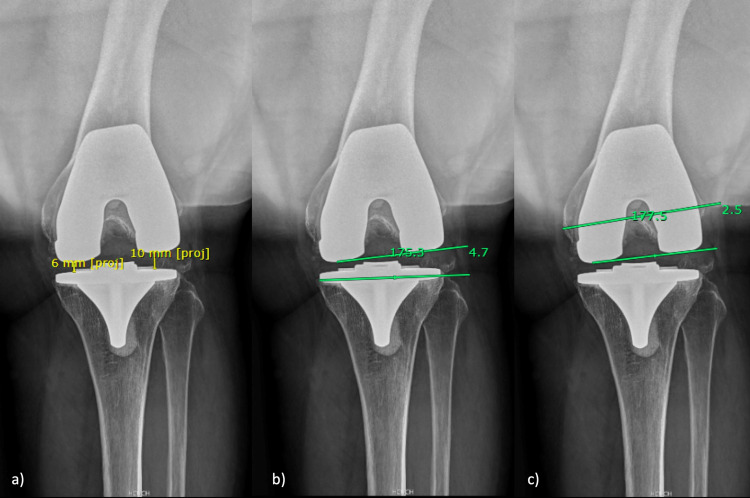
Seated view (a) 90º flexion lateral and medial joint space; (b) 90º flexion joint angle; (c) Femoral component rotation

Two distances were registered in millimeters between the most distal part of the femoral and the tibial components on the lateral and medial sides. Also, the side-to-side difference was calculated (AGflex) (Figure 4a).

Ninety Degree (90º) Flexion Joint Angle (fJA)

The angle was measured between a line that joins the most distal part of the medial and lateral femoral component and a line parallel to the tibial component. A positive value was registered when the angles opened laterally, and a negative value was registered when the angle opened to the medial side (Figure 4b). 

Femoral Component Rotation (FCR)

The angle was measured between a line that joints the center of the lateral epicondyle with the medial sulcus and a line that goes through the most distal part of the lateral and medial femoral component. A positive value was registered when the angles opened laterally, and a negative value was registered when the angle opened to the medial side (Figure 4c).

Lateral knee radiograph

Sagittal Anatomic Lateral View Femoral Component Alignment 1 (saLFA1)

**Figure 5:**
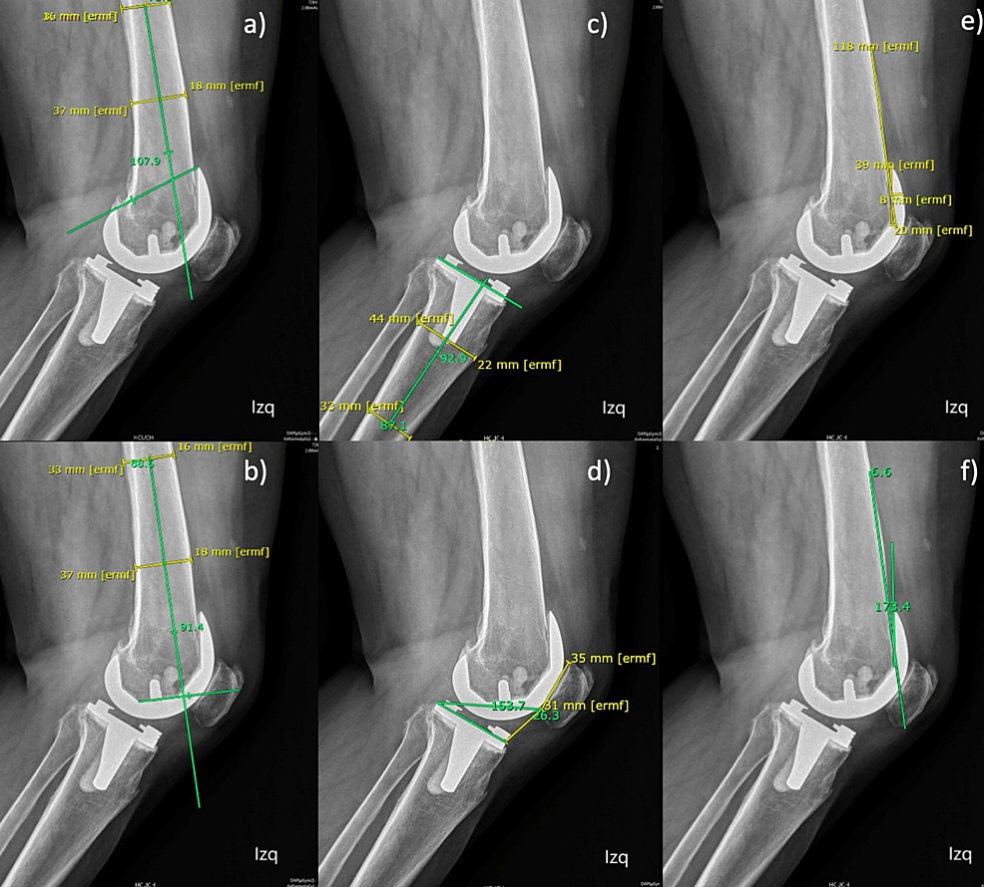
Lateral knee radiograph (a) Sagittal anatomic lateral view femoral component alignment 1; (b) Sagittal anatomic lateral view femoral component alignment 2; (c) Sagittal anatomic lateral view tibial component alignment; (d) Patela Height (yellow line). Patela Height 2 (green line); (e) Offset femoral anterior; (f) The anterior inclination angle of the femoral component

First, in the distal middle shaft of the femur, two lines were drawn from the anterior to the posterior cortex, and the middle of them was highlighted. The angle was measured with a line that goes through the middle of the two lines of the shaft and a line that goes through the most proximal parts of the anterior and posterior femoral components (Figure 5a).

Sagittal Anatomic Lateral View Femoral Component Alignment 2 (saLFA2)

First, in the distal middle shaft of the femur, two lines were drawn from the anterior to the posterior cortex, and the middle of those lines was highlighted. The angle was measured with a line that goes through the middle of the two lines of the shaft and a line that goes through the most distal axes of the femoral component (Figure 5b).

Sagittal Anatomic Lateral View Tibial Component Alignment (saLTA)

First, two lines were drawn from the proximal tibial shaft's anterior to the posterior cortex. The middle portion of both lines where highlighted. The angle was measured between a line that united both half distances of the lines drawn on the tibial shaft and a line parallel to the long axis of the tibial component. The posterior aspect of the angle was recorded (Figure 5c).

Patella Height (PH)

The proportion of the distance between the proximal to the distal patellar articular surface and the distance between the most distal part of the patellar articular surface to the most anterior-proximal part of the tibial component (Figure 5d).

Patella Height 2 (PH2)

The tibial-patella angle was measured (Figure 5d) [11].

Offset Femoral Anterior (Offset femoral)

The distance in the middle portion of the anterior aspect of the femoral component is between a line projected from the anterior femoral cortex to the most anterior part of the femoral component (Figure 5e).

The Anterior Inclination Angle of the Femoral Component (AIAF)

The angle was measured between a line parallel to the anterior cortex of the femur and a line parallel to the anterior axes of the femoral component (Figure 5f).

Axial knee radiograph

Patellar Lateralization Distance (PL)

**Figure 6:**
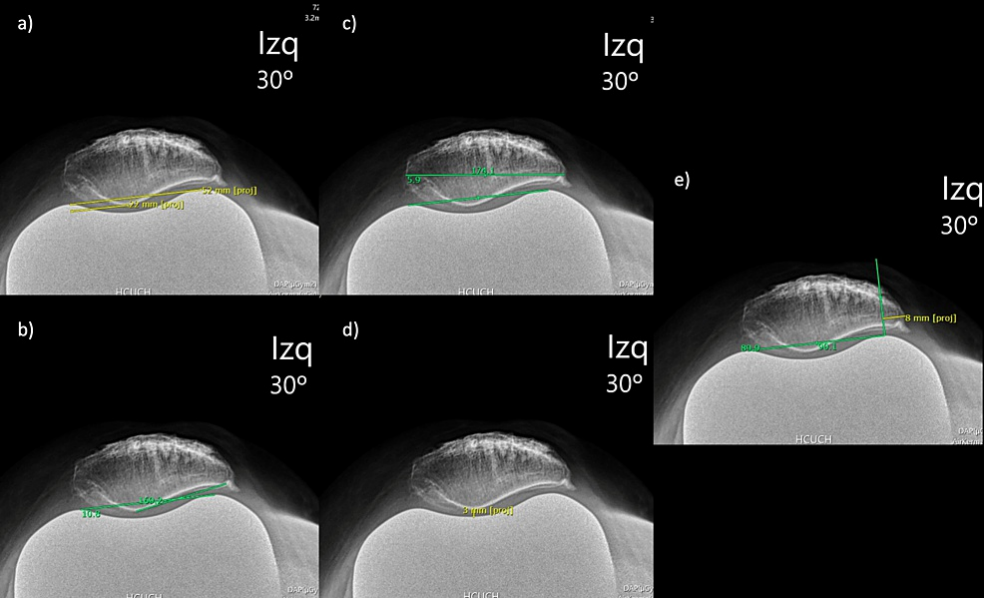
Axial knee radiograph (a) Patellar lateralization distance; (b) Opening lateral patellar angle; (c) Patellar tilt angle; (d) Patellofemoral joint space; (e) Lateral patellar overhanging

First, the mid-distance between the most anterior aspect of the lateral and medial femoral components was found. Then the distance between the apex of the patella and the mid-distance of the femoral component was recorded. A negative value indicates lateralization, and a positive distance medialization (Figure 6a). 

Opening Lateral Patellar Angle (OPT)

The angle was measured between a line that goes through the most anterior aspect of the lateral and medial side of the femoral component and a line that goes through the lateral facet of the patella (Figure 6b).

Patellar Tilt Angle (PT)

The angle was measured between a line that goes through the most anterior aspect of the lateral and medial side of the femoral component and a line that goes through the long axis of the patella (Figure 6c).

Patellofemoral Joint Space (pfJS)

The distance between the apex of the patella and the femoral component (Figure 6d).

Lateral Patellar Overhanging (LPO)

First, a line from the most anterior aspect of the lateral side of the femoral component and perpendicular to the line that unites the most anterior aspect of the medial and lateral parts of the femoral components was drawn. Then the distance between the perpendicular line to the most lateral aspect of the patella is measured (Figure 6e).

Two observers were recruited, a musculoskeletal radiologist (ME, observer #1) with eight years of experience in the field and a knee surgeon with six years of experience in the area (KS, observer #2). The interobserver agreement was estimated using the one-way random effects intraclass correlation coefficient (ICC). The ICC was interpreted using the lower 95% confidence interval limit as follows: below 0.50 poor; between 0.50-0.74: moderate; between 0.75 and 0.89: good; above 0.90: excellent [12]. All measurements were summarised in the median and interquartile range.

A linear regression was estimated for both observers to determine the relations of their HKA to the tibial and femoral coronal alignment. Pearson correlation was used to analyze those related radiologic parameters -if at least moderate concordance was achieved-that is, the sagittal alignment of the tibial (smTA, saTA, saLTA) and femoral component (smFA1, smFA2, saFA1, saFA2, saLFA1, and saLFA2), and patellar height (PH1 and PH2). Stata (StataCorp. 2021. Stata Statistical Software: Release 17. College Station, TX: StataCorp LLC) was used for statical analysis.

## Results

Full-length standing anteroposterior radiograph

The interobserver agreement of the LL and HKA was excellent; meanwhile, the agreement in the mLFA and mMTA was good and moderate, respectively (Table 1). The linear regression analysis in observer #1 was significant (p<0.001), achieving a B1 for mLFA of 0.90 [95% confidence interval (CI), 0.76 to 1.05)], (0 to -0.94) and a B1 for mMTA of -0.69 (95% CI, -0.85 to -0.52), with a constant (B0) of -18.96 (95% CI, -40.48 to 2.56). For Observer #2, the regression was also significant and estimated a B1 coefficient for mLFA of 1.05 (95% CI, 0.98 to 1.12) and -1.02 for mMTA (95% CI, -1.10 to -0.94) with a constant (B0) of -3.32 (95% CI, -13.60 to 6.97). The linear regression estimated for Observer #2 reached a greater R2 (R2=0.93) than for observer #1 (R2=0.69). Both models did not achieve homoscedasticity, p=0.015 and p=0.001, respectively. 

**Table 1:** Measurements and correlation of different views

	ME Radiologist	Knee Surgeon	Average difference	ICC	Quality
Right LL	752mm (50.56)	748mm (51.35)	4.0mm (±7.21)	0.99 (0.98 to 0.99)	Excellent
Left LL	753mm (50.84)	750mm (51.65)	2.9mm (±8.03)	0.98 (0.97 a 0.99)	Excellent
HKA	2.23° (3.64)	1.97° (3.64)	0.3° (±0.70)	0.98 (0.97 a 0.98)	Excellent
mLFA	91.6° (2.54)	91.8° (2.42)	0.1° (±1.33)	0.86 (0.81 a 0.90)	Good
mMTA	89.7° (2.25)	89.7° (2.14)	0.1° (±1.66)	0.72 (0.63 to 0.80)	Moderate
smFA1	75.0° (3.25)	74.4° (6.70)	0.7° (±6.71)	0.19 (0.02 to 0.35)	Poor
smFA2	0.2° (7.15)	6.94° (3.88)	6.8° (±8.69)	0 (0 to 0.17)	Poor
saFA1	74.2° (3.65)	74° (6.70)	0.2° (±7.08)	0.14 (0 to 0.31)	Poor
saFA2	0.51° (7.87)	6.9° (3.98)	6.4° (±9.52)	0 (0 to 0.17)	Poor
smTA	85.8° (3.06)	85.8° (3.00)	0.02° (±1.13)	0.93 (0.91 to 0.95)	Excellent
saTA	84.2° (3.34)	84.0° (3.17)	-0.2° (±1.76)	0.85 (0.81 to 0.90)	Good
eMJS	5.7mm (1.82)	5.9mm (1.85)	0.2mm (±0.44)	0.97 (0.96 to 0.98)	Excellent
eLJS	5.8mm (1.81)	6.0mm (1.74)	0.2mm (±0.41)	0.97 (0.96 to 0.98)	Excellent
AG ext	0.1 (0.54)	0.1 (0.62)	0.00mm (±0.47)	0.67 (0.58 to 0.77)	Moderate
eJA	0.1° (1.13)	0.2° (1.37)	0.1° (±1.82)	0 (0 to 0.17)	Poor
fMJS	5.4mm (2.50)	5.6mm (2.40)	0.1mm (±0.46)	0.98 (0.97 to 0.99)	Excellent
fLJS	6.6mm (2.92)	6.7mm (2.87)	0.1mm (±0.53)	0.98 (0.98 to 0.99)	Excellent
AG flex2	1.2 (2.22)	1.2 (2.31)	0.04mm (±0.66)	0.95 (0.94 to 0.97)	Excellent
fJA	1.3° (2.53)	0.3° (2.89)	- 1.0° (±3.49)	0.15 (0 to 0.31)	Poor
FCR	0.1° (2.75)	0.3° (2.17)	0.2° (±2.07)	0.65 (0.55 to 0.75)	Moderate
saLFA1	71.8° (3.99)	73.6° (3.54)	1.8° (±2.47)	0.70 (0.61 to 0.78)	Moderate
saLFA2	3.5° (2.87)	3.5° (3.03)	-0.04° (±1.15)	0.92 (0.89 to 0.95)	Good
saLTA	86.0 (7.22)	85.4 (7.00)	-0.6° (±2.68)	0.93 (0.90 to 0.95)	Excellent
PH	0.93 (0.16)	0.96 (0.14)	-0.03 (±0.10)	0.80 (0.75 to 0.87)	Good
PH2	30.5 (3.38)	29.6 (3.18)	0.9° (±1.43)	0.87 (0.83 to 0.91)	Good
Offset FA	7.9mm (3.15)	7.1mm (1.77)	-0.9mm (±2.96)	0.29 (0.13 to 0.45)	Poor
AIAF	8.4° (4.28)	8.4° (3.44)	-0.1° (±3.07)	0.69 (0.60 to 0.78)	Moderate
PL	-1.3mm (2.98)	-0.8mm (2.45)	0.4mm (±1.83)	0.77 (0.70 to 0.84)	Moderate
OPT	10.4° (4.98)	11.6° (3.38)	1.96° (±4.46)	0.64 (0.54 to 0.74)	Moderate
PT	7.7° (3.33)	6.8° (3.72)	-0.8° (±2.57)	0.71 (0.63 to 0.80)	Moderate
pfJS	1.6mm (1.00)	1.9mm (1.11)	0.3mm (±0.83)	0.63 (0.53 to 0.74)	Moderate
LPO	9.1mm (4.43)	7.1mm (3.53)	-2.13mm (±2.84)	0.69 (0.54 to 0.83)	Moderate

Full-length standing lateral radiograph

The interobserver agreement for all sagittal alignment of the femoral alignment achieved poor concordance. Meanwhile, on the tibial side, the mechanical sagittal alignment reached excellent concordance, and the anatomical alignment reached good concordance (Table 1). Also, each measurement got a significant and strong correlation for observer #1 (rho=0.95) and observer #2 (rho=0.93).

Anteroposterior standing knee view

Both joint space distances reached an excellent concordance. Meanwhile, the side-to-side difference and the joint line angle reached moderate and poor agreement, respectively (Table 1).

Seated view

The lateral and medial joint space distance and the side-to-side difference reached an excellent agreement. On the other hand, the joint line angle reached a poor agreement. The femoral component rotation achieved moderate agreement (Table 1).

Lateral view

The sagittal anatomical axis of the tibial component (saLTA) was the only measurement that reached an excellent concordance. The saLTA achieved a significant but poor correlation with both slope measurements - mechanical and anatomical- of the full-length lateral view, being 0.56 to mechanical and 0.52 to anatomical for observer #1 and 0.47 to mechanical and 0.49 to anatomical in observer #2.

Both patellar height measurements reached good concordance. The correlation between each measure was significant but moderate for observer #1 (rho=0.61) and observer #2 (rho= 0.71).

The femoral anatomical sagittal alignment using the most distal axes of the femoral component reached a good concordance (saLFA2). The other femoral anatomical parameter only reached a moderate agreement (saLFA1). The saLFA1 and 2 achieved a significant and strong correlation in observer #2 (rho=0.80) and moderate in observer #1 (0.71). The anterior inclination angle of the femoral component (AIAF) reached a moderate agreement, and the anterior femoral offset had a poor agreement (Table 1).

Axial knee view

All the patellar parameters had a moderate agreement. The PL reached an ICC of 0.77 but the lower limit of the 95% confidence interval was below 0.75, so it was interpreted as only moderate agreement. (Table 1).

## Discussion

In this study, a total of 32 radiological parameters were assessed for concordance, 10 (31%) of them achieved excellent, five (16%) "good", 10 (31%) "moderate", and seven (22%) "poor" concordance. The most important finding in this study is that at least one measurement in each radiograph view reached at least good concordance, except on the axial knee view. This means that a global alignment approach can be assessed to evaluate satisfaction or another outcome after TKA

Excellent concordance was achieved in the coronal limb alignment (HKA). HKA is the most studied radiological parameter in the literature, being still controversial about the best desirable alignment - mechanical, anatomical, functional, or kinematic [13, 14]. Nevertheless, most of those studies miss the alignment in the sagittal and axial planes. A recent study reported that lack of satisfaction after TKA was strongly associated with femoral component extension or flexion of more than 10 degrees and a tibial slope of five degrees than in the native joint of the patient [15]. In this study, two measurements of the sagittal alignment of the tibial component achieved excellent concordance (lateral long limb view, SMTA, and lateral knee view, saLTA). Still, those measurements reached just a moderate correlation in-between them, making it difficult to recommend one measure above the other. We think it is better to obtain both for a complete radiological evaluation of TKA. On the contrary, a recent report [16] shows a strong correlation between the mechanical axis in a lateral long-leg view and with anatomical axis in a lateral view; nevertheless, in the study, the measurement was made from the midpoint of the distal end of the tibia, which allows them a more comprehensive view of the tibia than we had using a regular lateral knee view. As all femoral measurements in the lateral long limb view had poor concordance, for future assessment, we will use a lateral leg view in the standing position. This will allow us to measure the mechanical sagittal axis of the tibial component, as this view is easier to acquire and has a lower cost.

One measurement of the sagittal alignment of the femoral component (lateral knee view, saLFA2) accomplished an ICC of 0.92, good enough for excellent. Still, the lower limit of the 95% interval confidence was 0.89, so the quality was just "Good". An excessive flexion of the femoral component is a risk factor for flexion contractures [17], but on the other hand, an excessive extension of the femoral component has been associated with anterior knee pain [18] 

It is remarkable that imageless robotic-assisted TKA, as ROSA (Zimmer®), estimates and does the planning using the mechanical sagittal alignment, not the anatomical. So, understanding the difference between mechanical and anatomical sagittal alignment is needed to agree on what was planned in robotic surgery and the final result [19]. 

Also, excellent agreement was accomplished in the medial and lateral gap in extension and flexion. Flexion instability after total knee arthroplasty (TKA) has been related to an increased flexion gap compared to the extension gap [20].

Femoral rotation in the seated view has been reported to have a better concordance in other studies [7-8]. We will re-evaluate the acquisition technique of the radiograph and the identification of the epicondyles for further studies. This could make it possible to reach a better agreement and make more appropriate the evaluation of the axial alignment of the TKA at a lower cost than TC. Similar efforts should be made to assess radiological parameters on the axial patella view. Patellar lateralization seems to be the more reliable measurement as it achieved 0.77 ICC and only a 0.4 mm average of the difference. This measurement has been reported to be associated with the axial alignment of the femoral component [21]. Although only moderate concordance measures were obtained in the knee axial view, we consider that this view should be kept within the TKA follow-up radiological set, as it is an acquisition with which most centers are familiar and the association has been found between measures in this view and patients reported outcomes [7-22]. The latter has been described in TKA with patellar replacement, unlike this study in which the patella was retained which may explain the difference in agreement of the measurements.

Different methods have been reported to measure patellar height after TKA [23]. In this study, we evaluate two with good concordance but moderate correlations. This is concordant with other studies [11, 23], so we recommend for future studies consistently perform at least two measurements of the patellar height. 

We propose incorporating a complete evaluation after surgery to determine the global alignment related to good results. It is important to remember that the measurement of the angles on our patients' follow-up is a part of the systematic evaluation and is useful for a better global understanding. Khalifa et al. [24], proposed a similar assessment after TKA but did not report concordance in the measurement, which is crucial to obtain reliable results. 

The limitation of this study is that only two raters were recruited. Nevertheless, both are specialists in their fields. Also, this measurement is only validated for a Vanguard CRAS type of arthroplasty, as the prosthesis design can make the radiologic parameter more or less accurate. 

## Conclusions

The additional radiograph views assessed in this study, including the full-length standing lateral radiograph of the lower extremity, the patellar axial view with the knee flexed at 30º, and the seated view, may provide valuable information for assessing alignment on total knee arthroplasty, complementing routine radiographs set for knee replacement follow-up in many institutions. These views may help to identify potential causes of patient dissatisfaction and provide a more complete picture of alignment, which may ultimately lead to better outcomes for patients undergoing knee arthroplasty. So, it is imperative to use coronal, sagittal, and axial alignment evaluation to correlate with functional outcomes and survival after TKA.

Excellent and good agreement on different views of the knee to assess results after TKA can be achieved when observers are experienced and knowledgeable in the field.
